# Climate overrides prey availability in shaping the decadal population dynamics of two sympatric lady beetles in monsoon farmlands of central China

**DOI:** 10.7717/peerj.21748

**Published:** 2026-09-29

**Authors:** Mengyuan Du, Yanhong Hou, Xiaoxin Song, Li Chen, Zhiye Fan, Wenhao Wang, Di Liu, Shimin Li, Jianrong Huang, Qi Chen

**Affiliations:** 1 Luohe Academy of Agricultural Sciences, Luohe, Henan, China; 2 Yancheng Observation and Research Station, Ministry of Agriculture and Rural Affairs, Luohe, Henan, China; 3 Plant Protection Research Institute, Henan Academy of Agricultural Sciences, Zhengzhou, Henan, China; 4 No. 0 Entomological Radar Field Scientific Observation and Research Station of Henan Province, Zhengzhou, Henan, China; 5 Xinxiang Observation and Research Station, Ministry of Agriculture and Rural Affairs, Xinxiang, Henan, China

**Keywords:** *Harmonia axyridis*, *Propylea japonica*, Long-term monitoring, Climatic factors, Predator-prey decoupling, Conservation biological control

## Abstract

**Background:**

Under global warming, insect responses vary across regions, yet the population trends of dominant lady beetle species in central China remain poorly understood. Whether abiotic forcing (climate) or biotic interactions (prey availability) dominates their long-term population dynamics is unresolved.

**Methods:**

We monitored lady beetle populations using black-light traps during March–November, 2015–2024, at a fixed farmland site in Luohe, Henan, China. Trap data were integrated with local meteorological records and aphid catches from a suction trap.

**Results:**

A total of 21,572 individuals were collected, comprising *Propylea japonica* (10,511) and *Harmonia axyridis* (11,061). *P. japonica* showed pronounced interannual variation (range: 65–4,258 individuals) and exhibited three seasonal peaks in early June, mid-July, and late September. Its first capture date advanced significantly (−3.5 d yr^−1^, *P* = 0.045), whereas *H. axyridis* phenology remained stable, generating clear temporal niche partitioning (*P. japonica* dominant June–July; *H. axyridis* dominant August–October). *H. axyridis* catches increased significantly over the decade (*R*^2^ = 0.69, *P* = 0.0028). Stepwise regression identified May precipitation as a shared positive predictor for both species, while temperature effects were species-specific: June temperature was a positive predictor for *H. axyridis*, and previous-November temperature was a positive predictor for *P. japonica* (both models explaining >90% of interannual variance). Aphid catches showed no positive association with either predator; predator peaks lagged behind aphid peaks by ~48 days in spring, and summer predator maxima coincided with the seasonal aphid trough.

**Conclusions:**

Climatic factors, rather than prey availability, are the dominant correlates of population dynamics for these two species, which respond asymmetrically to shared climatic drivers. The decoupling of predator activity from aphid densities indicates a limited numerical response, suggesting that abiotic forcing can outweigh trophic interactions in this monsoon-governed system. Conservation biological control should integrate long-term climatic monitoring rather than relying solely on prey-driven paradigms.

## Introduction

Global warming is driving pronounced shifts in the distribution, phenology, and population dynamics of insects, with cascading effects on the ecosystem services they provide (Parmesan & Yohe, 2003; Walther et al., 2002). In agricultural landscapes, predatory natural enemies are particularly sensitive to climatic fluctuations, yet their responses remain highly variable across regions and taxa (Thomson, Macfadyen & Hoffmann, 2010). Lady beetles (Coleoptera: Coccinellidae) are among the most important aphidophagous predators, and their capacity to suppress pest populations is closely tied to both climatic conditions and prey availability (Dixon, 2000; Hodek, van Emden & Honěk, 2012). A central, unresolved question in agroecology is whether prey-driven population regulation or climate-driven constraints dominates the long-term dynamics of predator populations (Menge & Sutherland, 1987; Boukal et al., 2019). While long-term monitoring programs in Europe and North America have documented substantial climate-driven shifts in coccinellid communities (Comont et al., 2014; Honěk et al., 2017), equivalent knowledge from central China—a region governed by the East Asian monsoon with a summer rainfall–temperature regime fundamentally different from temperate systems—remains exceptionally scarce. Understanding how dominant lady beetle species in this region respond to long-term climatic changes is therefore critical for predicting the stability of conservation biological control under future climate scenarios.

The two most prevalent lady beetles in the farmlands of central China are the native *Propylea japonica* and the cosmopolitan *Harmonia axyridis*. Both species are voracious aphid predators and frequently dominate the natural enemy community in wheat, maize, and vegetable fields (Koch, 2003; Yang et al., 2018; Hodek, van Emden & Honěk, 2012). Despite their sympatry and overlapping prey spectra, they differ markedly in key ecological traits: *H. axyridis* is a large, highly polyphagous and dispersive predator capable of tracking prey across heterogeneous landscapes, whereas *P. japonica* is a smaller, early-season specialist that typically shows close temporal coupling with aphid outbreaks in cereal crops (Hodek, van Emden & Honěk, 2012; Osawa, 2000). However, whether this presumed predator–prey coupling persists under long-term climatic shifts in natural agroecosystems remains untested. These intrinsic biological differences suggest that the two species may not only respond asymmetrically to climatic forcing, but also partition the growing season into distinct temporal niches, thereby reducing direct interspecific competition while differentially tracking prey phenology. Laboratory and short-term field studies have confirmed that temperature and precipitation strongly influence their development, survival, and reproductive output (Atlihan & Chi, 2008; Hodek, van Emden & Honěk, 2012). However, in natural agroecosystems, climatic factors and prey availability are deeply intertwined. Extreme temperatures and shifting precipitation regimes can disrupt trophic linkages, decouple predator–prey dynamics, and constrain the numerical response of predators even when prey is abundant (Durant et al., 2007; Hance et al., 2007; Both et al., 2006). Indeed, under certain climatic regimes, predator abundance may even become negatively correlated with prey density, reflecting a complete breakdown of numerical response (Dixon, 2000; Hemptinne, Dixon & Coffin, 1992). Whether such abiotic override of trophic interactions occurs in the monsoon-influenced farmlands of central China, and whether it manifests differently in species with contrasting life-history strategies, remains entirely unknown.

Despite the ecological and agricultural significance of *P. japonica* and *H. axyridis*, several critical knowledge gaps persist. First, most existing studies are based on short-term surveys or laboratory experiments, which cannot capture interannual variability in population sizes, gradual phenological trends such as shifts in first appearance dates, or the progressive decoupling of predator–prey synchrony over years. Second, the contrasting population dynamics and seasonal succession patterns of these two species have rarely been examined within a unified, multi-year framework that simultaneously integrates overwintering conditions, growing-season precipitation, and concurrent aphid monitoring. Moreover, whether the population trajectories of these two species have diverged over the past decade—and what this implies for community restructuring under warming—has not been documented. Third, it remains unknown whether warming has differentially advanced the spring phenology of the early-season specialist *P. japonica* relative to the generalist *H. axyridis*, or whether the peak of predator activity has disconnected from the peak of aphid population-potentially reflecting a limited numerical response under low prey densities. Additionally, the multi-peak seasonal activity pattern reported for *P. japonica* in short-term studies has never been characterized across a decadal timescale (Gao, Li & Zhao, 2006), leaving its interannual stability and climatic sensitivity unknown. Addressing these gaps requires a decadal-scale, multi-species dataset that pairs standardized predator trapping with detailed meteorological records and pest monitoring—an approach that has been advocated but rarely realized (Harrington et al., 2007).

To fill these gaps, we conducted a 10-year (2015–2024) fixed-site field study in a representative agricultural landscape in Luohe, Henan, central China. Adult lady beetles were sampled using black-light traps (Southwood & Henderson, 2000), aphid abundance was monitored with a suction trap (Macaulay, Tatchell & Taylor, 1988), and these records were integrated with local meteorological data. We analysed interannual population dynamics, seasonal phenology, temporal niche partitioning, and first capture dates of *P. japonica* and *H. axyridis*. Specifically, we tested three hypotheses: (1) climatic variables, rather than prey availability, are the dominant correlates of population fluctuations for both species, with the identity, strength, and direction of key predictors differing asymmetrically between species; (2) climate warming has differentially altered the phenology of the two species, which exhibit temporal niche partitioning across the growing season; and (3) predator peaks lag behind aphid peaks, positive predator–prey correlations are absent at both annual and seasonal scales, and the numerical response of predators to prey availability is limited. Our study provides one of the first decadal assessments of dominant lady beetle responses to climate change in the monsoon-influenced farmlands of central China, offering mechanistic insights for conservation biological control strategies that should integrate long-term climatic trends—rather than relying solely on prey-driven paradigms—to sustain effective natural enemy communities.

## Materials and Methods

### Study site

This study was conducted at a fixed monitoring site within the Luohe Academy of Agricultural Sciences, Luohe City, Henan Province, central China (33°36′N, 113°59′E; elevation 60 m). The monitoring plot encompasses approximately 28.33 ha and is embedded in a typical wheat–maize rotation landscape characteristic of the Huang-Huai-Hai Plain. Adjacent fields are intermittently planted with soybean, peanut, sesame, and sweet potato. The surrounding area is dominated by cropland, field margins, and scattered non-crop vegetation (Fig. 1). The region experiences a warm-temperate semi-humid monsoon climate with a mean annual temperature of approximately 15.2 °C and mean annual precipitation of approximately 788 mm, with >60% of rainfall concentrated in June–August.

**Figure 1:**
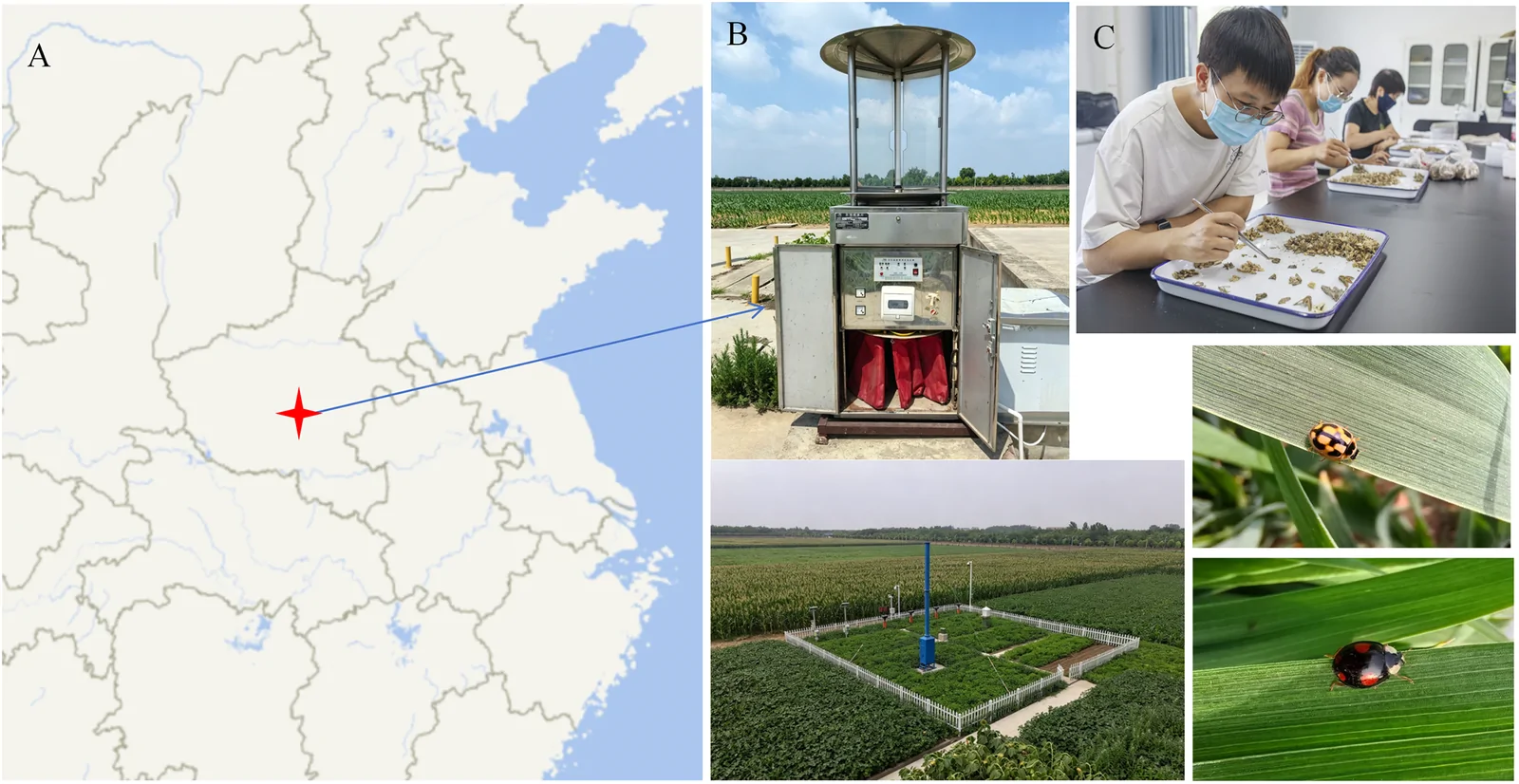
Location of the monitoring site and schematic diagram of the monitoring system. (A) Geographic location of Luohe City, Henan Province, China. (B) Field placement of the black light trap and suction trap. (C) Workflow of sample collection, preservation, sorting and identification.

### Insect sampling

#### Lady beetle monitoring

Adult lady beetles were sampled using a single black-light trap (model JBD2; Hebi Jiaduo Technology Co., Ltd., Hebi, China) equipped with a 20-W ultraviolet lamp (peak wavelength 365 nm). The lamp centre was positioned 1.5 m above ground level. The trap incorporated an automated time-control system governing the nightly on/off cycle. Phototactic insects contacting the internal baffle fell into a collection chamber, where they were killed by far-infrared radiation and accumulated in a removable collection bag at the base.

The light trap was operated continuously from March through November each year from 2015 to 2024. Captured insects were retrieved every morning, transferred to the laboratory, and stored at −20 °C for short-term preservation. Adult lady beetles were sorted from the total catch and identified to species level following standard taxonomic keys (Ren et al., 2009; Yu, 2010). Specimens with damaged or ambiguous diagnostic features were re-examined by an experienced entomologist; those that could not be confidently assigned to species were excluded. The same trap unit, lamp type, placement, and identification protocol were maintained throughout the 10-year period. No spatially replicated light traps were deployed in this long-term fixed-site survey; analyses were therefore based on temporal replication across sampling dates and years.

#### Aphid monitoring

Airborne aphids were sampled using a suction trap (model Keyun ST-1B; Jiaozuo Baiyun Industrial Co., Ltd., Jiaozuo, China). The trap tower was 8.3 m in height with an airflow rate of 2,000 m^3^ h^−1^. The suction trap operated 24 h day^−1^ from March through November each year. Aphid samples were collected daily, sorted, and counted. Total aphid catches (all species combined) was used as an index of prey availability.

### Meteorological data

Daily meteorological records (mean air temperature, relative humidity, and precipitation) were obtained from the Luohe Meteorological Service, China Meteorological Administration, located approximately 8 km from the monitoring site. Daily values were aggregated into monthly means (temperature and relative humidity) and monthly totals (precipitation). The wintering period refers to the period from November of the previous year to March of the current year.

### Data processing

Daily lady beetle catches and aphid counts were aggregated into dekads (10-day periods: days 1–10, 11–20, and 21-end of month) to reduce short-term stochastic noise from daily weather fluctuations and minor variation in trap efficiency. Light-trap catches were interpreted as a standardized index of relative adult activity–abundance under light-trap conditions, not as a direct estimate of absolute field population density (Southwood & Henderson, 2000). For each species and year, the following metrics were derived: Annual light-trap catch: total number of adults captured from March to November.

First capture date (FCD): the calendar date of the first adult capture.

Final capture date: the calendar date of the last adult capture.

Occurrence period: the number of days between the first and final capture dates.

Dekadal catch peak: a local maximum in 10-day catches that exceeded catches in both the preceding and the subsequent 10-day interval. Monthly totals of lady beetle catches and aphid counts were used to examine associations with meteorological variables and to characterize predator–prey temporal relationships. Rarely captured coccinellid species were recorded for community composition; however, long-term population analyses were restricted to the two numerically dominant species, *H. axyridis* and *P. japonica*.

### Statistical analysis

All statistical analyses were performed in R version 4.3.1 (R Core Team, 2023) with α = 0.05. Count data were log-transformed [ln(N + 1)] where necessary to satisfy model assumptions; regression models in § 2.5.3 used z-score standardization instead (see below). Results are reported with 95% confidence intervals where appropriate.

#### Interannual trends and phenological shifts

Linear regression was used to test for temporal trends in annual catches and in first capture dates over the 10-year period. Differences in phenological trends between the two species were assessed by testing the difference in regression slopes. The interannual trend in total aphid abundance was likewise evaluated by linear regression on annual aphid catches.

#### Seasonal activity patterns and temporal niche partitioning

Dekadal catches were averaged across years to characterize the mean seasonal activity profile of each species. The timing and magnitude of seasonal peaks were identified from the dekadal catch curves. To describe temporal niche partitioning, the relative dominance of each species was calculated for each dekad as its share of the combined catch of the two lady beetle species, using dekad totals pooled across all study years (*i.e*., summing the catch of each species over years within a dekad before dividing). Periods in which one species consistently exceeded 50% of the combined catch were designated as its dominance window. The seasonal succession pattern was further visualized by plotting monthly species proportions across the March–November monitoring period.

#### Climatic predictors of annual catches

Separate multiple linear regression models were constructed for *H. axyridis* and *P. japonica*. To place the two species on a common scale, the response variable (standardized annual catch) was expressed as z-scores, so that coefficients are directly comparable between species, each unit change representing a shift in annual catch in standard-deviation units. Candidate predictors comprised monthly mean temperature, total precipitation, and mean relative humidity for the twelve consecutive months from the preceding November through the current October; this window ends 1 month before the close of the trapping season to avoid simultaneity with the response. Predictors were selected by bidirectional, Akaike information criterion (AIC)-based stepwise regression. Model fit was summarised with adjusted *R*^2^ and in-sample Root Mean Squared Error (RMSE) on the original catch scale.

These models are exploratory and reported as in-sample descriptive fits only. With *n* = 10 and stepwise selection over a 36-candidate predictor pool, *R*^2^ reflects in-sample fitting rather than generalisation; we therefore make no claim of predictive performance and interpret retained coefficients as candidate climatic windows co-varying with interannual catch.

#### Predator–prey temporal relationships and synchrony

The main aphid peak and the corresponding lady beetle peak were identified for spring (April–June) and autumn (September–October) each year from dekadal catch data. Within each season and year, the lag was calculated as the lady beetle peak date minus the aphid peak date (in days); a season–year with an undetectable peak yielded no lag. Spring and autumn lags were compared per species, with a paired t-test (spring *vs*. autumn within each year) for *H. axyridis* and an unpaired Welch’s t-test for *P. japonica*. Summer peaks (July–August) were described qualitatively. For each species, annual aphid totals and annual lady beetle catches were correlated using Spearman’s rank correlation, or Pearson’s where the Shapiro–Wilk test indicated normality.

#### Interpretive caveats

Given the observational nature of the dataset and limited temporal replication (*n* = 10 years), all regression and correlation analyses are interpreted as exploratory, identifying statistical associations rather than causal mechanisms. In particular, the stepwise-selected models are regarded as identifying plausible climatic correlates rather than definitive predictors.

## Results

### Species composition and interannual population trends

Over the 10-year monitoring period (2015–2024), black-light trapping recorded six coccinellid species: *H. axyridis*, *P. japonica*, *Coccinella septempunctata*, *Hippodamia variegata*, Calvia muiri, and Chilomenes quadriplagiata. Of these, only *H. axyridis* and *P. japonica* were enumerated (21,572 individuals: 11,061 and 10,511, respectively) and designated the dominant species for subsequent quantitative analyses. The remaining four occurred only sporadically and were excluded from the interannual-trend and seasonal-phenology analyses.

The annual light-trap catches of *H. axyridis* increased significantly over the decade (*R*^2^ = 0.69, *P* = 0.0028; Fig. 2), rising from a low of 379 individuals in 2015 to a peak of 2,173 in 2022. In contrast, *P. japonica* exhibited pronounced interannual fluctuations without a consistent directional trend, with annual catches ranging from 65 individuals in 2019 to 4,258 in 2023. Despite these contrasting trajectories, the annual catches of the two species were significantly positively correlated (Pearson r = 0.72, *P* = 0.019), indicating that years favourable for one species tended to be favourable for the other.

**Figure 2:**
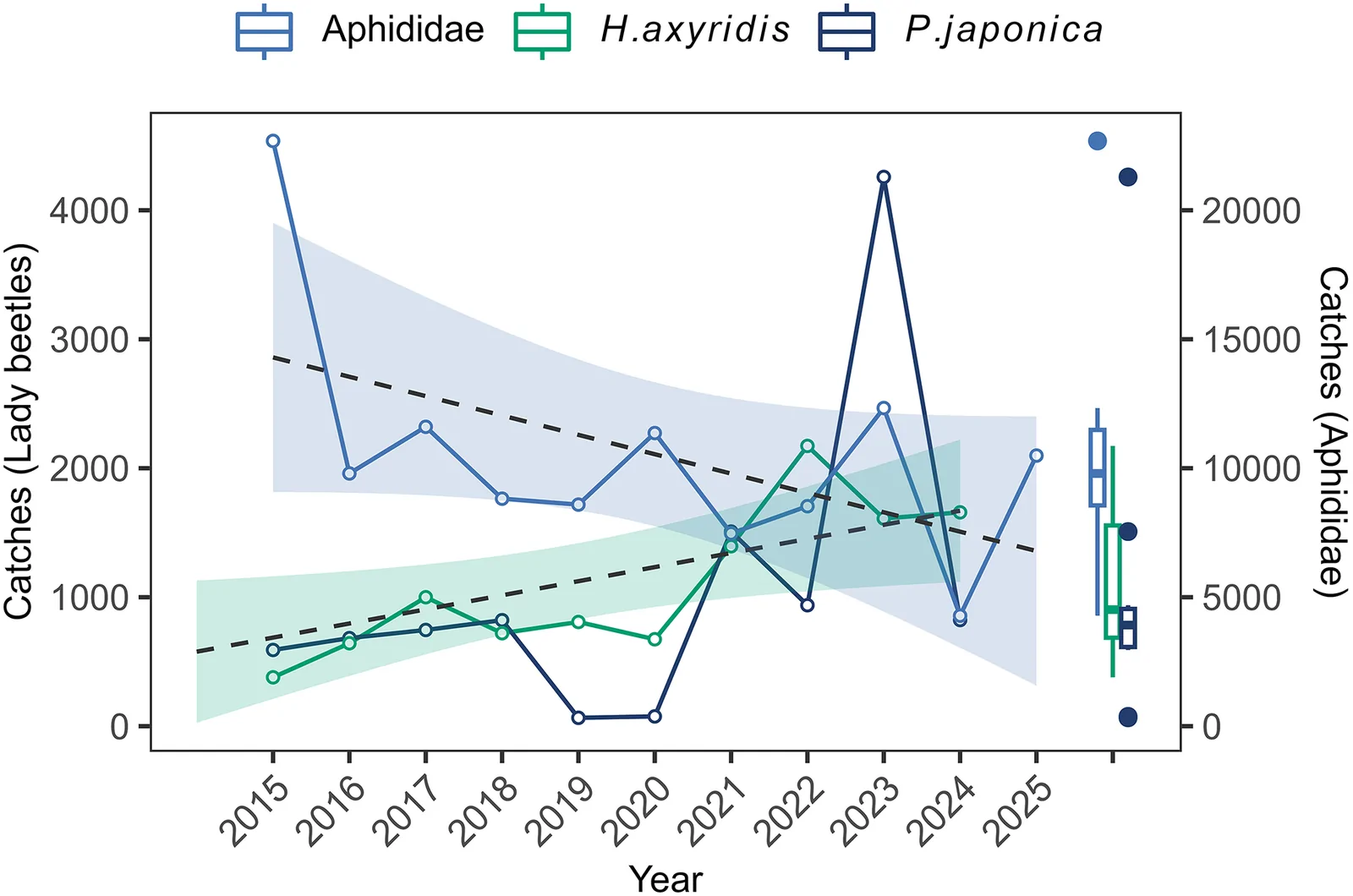
Interannual variation in annual catches of *Harmonia axyridis*, *Propylea japonica*, and aphids, 2015–2024. Catches of the two lady beetle species are read on the left y-axis and aphid catches on the right y-axis. Points connected by lines represent annual catches. Linear regression lines (dashed) with 95% confidence intervals (shaded bands) are shown for *H. axyridis* (increasing) and for aphids (decreasing); no regression line is fitted for *P. japonica* because its annual catches showed no consistent directional trend. Box plots on the right summarize the distribution of the 2015–2024 annual catches for each taxon.

Annual aphid catches, recorded by the suction trap, varied substantially among years, ranging from 4,283 individuals in 2024 to 22,688 in 2015. Linear regression revealed a non-significant declining trend (slope = −750.6 individuals year^−1^, *P* = 0.086; Fig. 2), indicating that prey availability showed no statistically robust long-term decline over the study period. Accordingly, annual predator catches showed no positive association with annual aphid catches (Spearman’s ρ = −0.26, *P* = 0.47 for *P. japonica*; ρ = −0.56, *P* = 0.09 for *H. axyridis*), indicating a limited interannual numerical response of predators to prey availability.

### Phenological shifts and seasonal activity patterns

#### First and final capture dates and occurrence periods

Monitoring of first capture dates (FCD) revealed divergent phenological trends between the two dominant species (Table 1). The FCD of *H. axyridis* ranged from mid-March to late April and showed no significant temporal trend over the decade (*P* = 0.97). In contrast, the FCD of *P. japonica* advanced significantly (slope = −3.50 d yr^−1^, *P* = 0.045), shifting from late May in 2015 to early April in 2024. On average, *H. axyridis* appeared 16.8 ± 21.6 d earlier than *P. japonica*. Final capture dates for both species fell between late September and mid-November, with no directional shift in either species. The mean occurrence period was 198.2 ± 16.7 d for *H. axyridis* and 181.8 ± 21.7 d for *P. japonica*. Over the decade, occurrence periods showed a non-significant increasing trend in both species (*H. axyridis*: slope = +1.38 d yr^−1^, *P* = 0.48; *P. japonica*: slope = +3.50 d yr^−1^, *P* = 0.15).

**Table 1:** First and final capture dates and occurrence periods of *Harmonia axyridis* and *Propylea japonica*, 2015–2024.

Year	*Harmonia axyridis*			*Propylea japonica*		
	First capture date	Final capture date	Occurrence period (d)	First capture date	Final capture date	Occurrence period (d)
2015	25 Apr.(2)	21 Oct.(2)	179	28 May(1)	23 Oct.(2)	148
2016	13 Apr.(2)	19 Oct.(2)	189	30 Apr.(3)	19 Oct.(4)	172
2017	4 Apr.(1)	14 Oct.(1)	193	14 Apr.(1)	9 Oct.(2)	178
2018	24 Mar.(1)	24 Oct.(4)	214	14 May(16)	14 Oct.(2)	153
2019	18 Apr.(2)	22 Oct.(2)	187	18 Apr.(1)	11 Nov.(1)	207
2020	19 Mar.(1)	8 Oct.(1)	203	2 May(1)	15 Nov.(1)	197
2021	12 Apr.(1)	14 Nov.(1)	216	12 Apr.(1)	14 Nov.(2)	216
2022	20 Mar.(1)	31 Oct.(1)	225	11 Apr.(1)	7 Oct.(1)	179
2023	18 Apr.(2)	6 Nov.(2)	202	29 Apr.(1)	6 Nov.(2)	191
2024	25 Apr.(1)	16 Oct.(1)	174	5 Apr.(1)	29 Sep.(7)	177

**Note:**

Values in parentheses are the numbers of individuals trapped on the respective first and final capture dates.

#### Seasonal activity profiles

Dekadal mean catch profiles, averaged over the 10-year study period, revealed distinct seasonal activity patterns for the two species (Fig. 3). *H. axyridis* adults were active continuously from late May through late September, exhibiting a sustained, multi-peak phenology (Fig. 3A). Rather than a single sharp maximum, the mean profile (black line) formed a broad plateau from late July to mid-August, with a smaller secondary rise in late September; the timing of the annual maximum nonetheless varied considerably among years (*e.g*., a late-May peak in 2017 (Fig. 3A)). Catches were thus relatively evenly spread across the active season, with no single dekad accounting for more than 23% of any year’s total.

**Figure 3:**
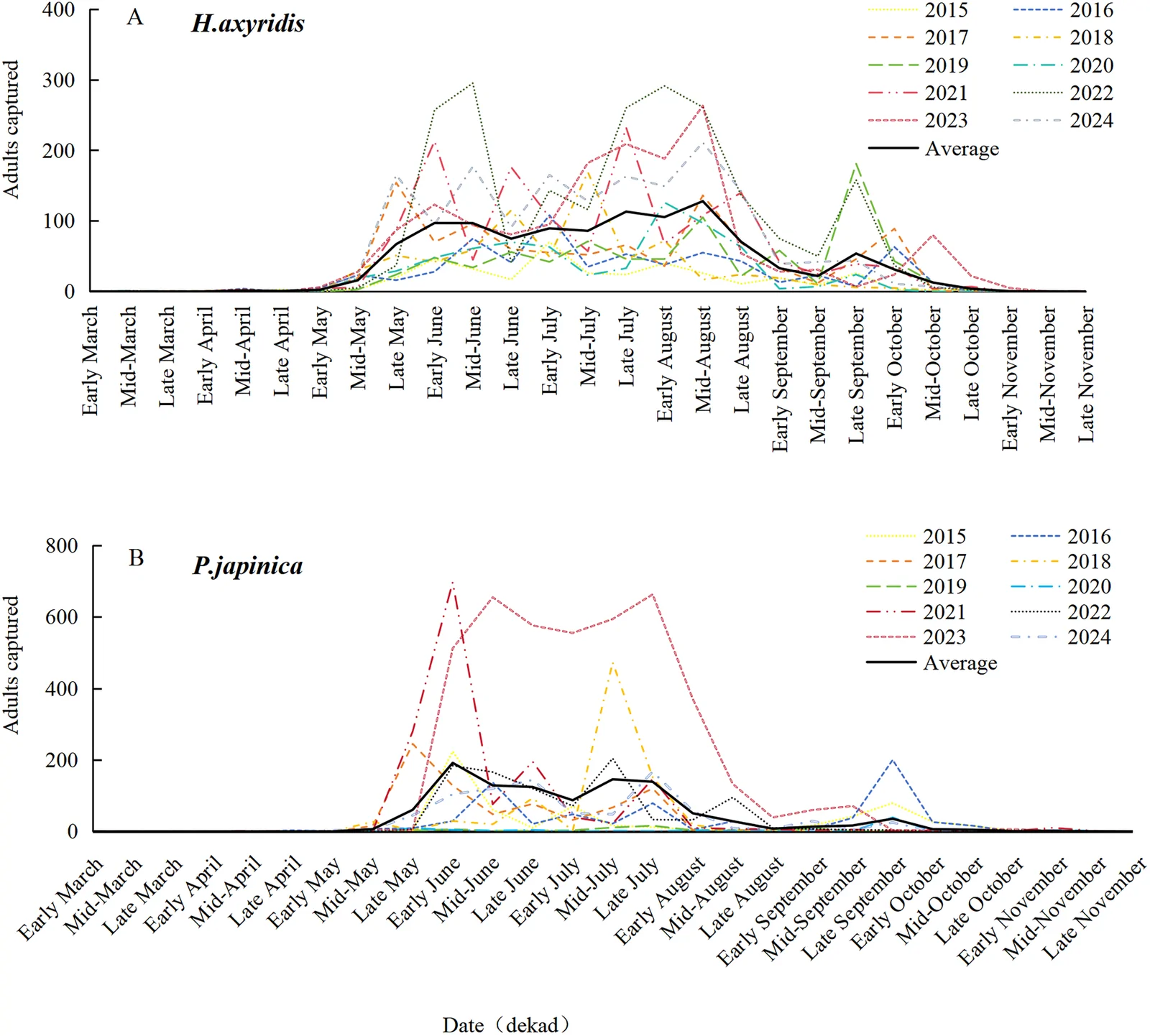
Dekadal seasonal activity profiles of *Harmonia axyridis* (A) and *Propylea japonica* (B), 2015–2024. Coloured lines are individual years (2015–2024); the black line (legend: Average) is the mean of per-year dekadal catches; Y-axis: adults captured per dekad.

In contrast, *P. japonica* displayed a more pulsed phenology (Fig. 3B). The mean profile showed three main seasonal peaks—in early June, mid-July, and late September—with the highest mean dekadal catch in early June. Peak number and timing, however, varied strongly among years, ranging from a single weak peak (2019, 2020) to three discrete peaks or a sustained plateau (Table 2). The year 2023 was exceptional: catches remained above 500 individuals per dekad from early June through late July, forming a prolonged period of high catches rather than a discrete peak (Table 2).

**Table 2:** Interannual variation in peak periods and maximum dekadal catches of *Propylea japonica*, 2015–2024.

Year	Number of peaks	Main peak period(s)	Maximum dekadal catch	Notes
2015	Three	Early June; early July; late September	224	Three separated peaks
2016	Three	Mid-June; late July; late September	200	Late September peak was prominent
2017	Two	Late May; late July	245	Earlier first peak
2018	Two	Late June; mid-July	473	Mid-July peak accounted for 58% of annual catch
2019	One	Late July	16	Lowest annual catch
2020	One	Late September	39	Weak seasonal peak
2021	Three	Early June; late June; late July	697	Strong early-June peak
2022	Three	Early June; mid-July; mid-August	204	Multiple moderate peaks
2023	Sustained peak	Early June-late July	>500 per dekad	Prolonged high catches period
2024	Three	Early June; late June; late July	167	Peaks concentrated in early summer

**Note:**

Number of peaks and main peak period(s) were read from each year’s dekadal catch curve (Fig. 3B); “maximum dekadal catch” is the largest single-dekad total in that year.

#### Temporal niche partitioning

Analysis of catch proportions across dekads demonstrated clear temporal niche partitioning between the two species (Fig. 4). *P. japonica* dominated light-trap catches from early June to late July, with its proportional contribution peaking at 66.4% in early June. Conversely, *H. axyridis* became the dominant species from early August to late October, coinciding with the decline of *P. japonica* to a minimum of 9.9% in late August. Prior to late May, *H. axyridis* accounted for nearly all captures, reflecting the delayed emergence of *P. japonica*. This complementary seasonal succession established two distinct dominance windows within the growing season.

**Figure 4:**
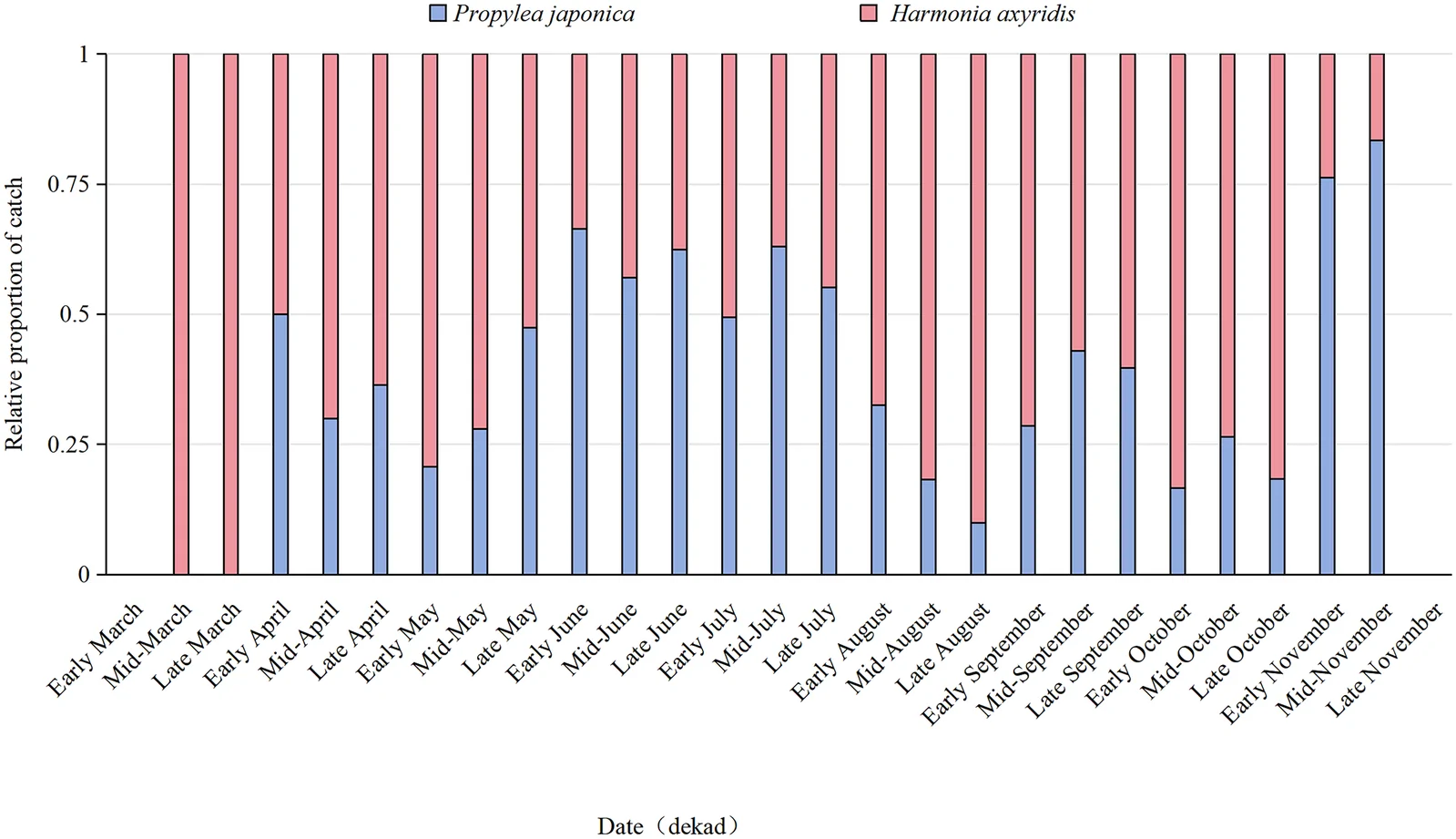
Dekadal (10-day) proportional composition of light-trap catches for the two lady beetle species, *Propylea japonica* and *Harmonia axyridis*. Bars are stacked to one; each segment is one species’ share of that dekad’s year-pooled catch.

### Climatic correlates of population dynamics

#### Correlation analysis

Spearman rank correlations between population metrics and meteorological variables revealed species-specific climatic associations (Fig. 5). For *H. axyridis*, annual catch was positively correlated with overwintering temperature (previous-November mean) and current-year June mean temperature, as well as with the mean temperature of April–July, and negatively correlated with June precipitation. The FCD of *H. axyridis* was negatively correlated with January humidity and rainfall. For *P. japonica*, annual catch was significantly negatively correlated with June precipitation, and its FCD was negatively correlated with previous-November mean temperature.

**Figure 5:**
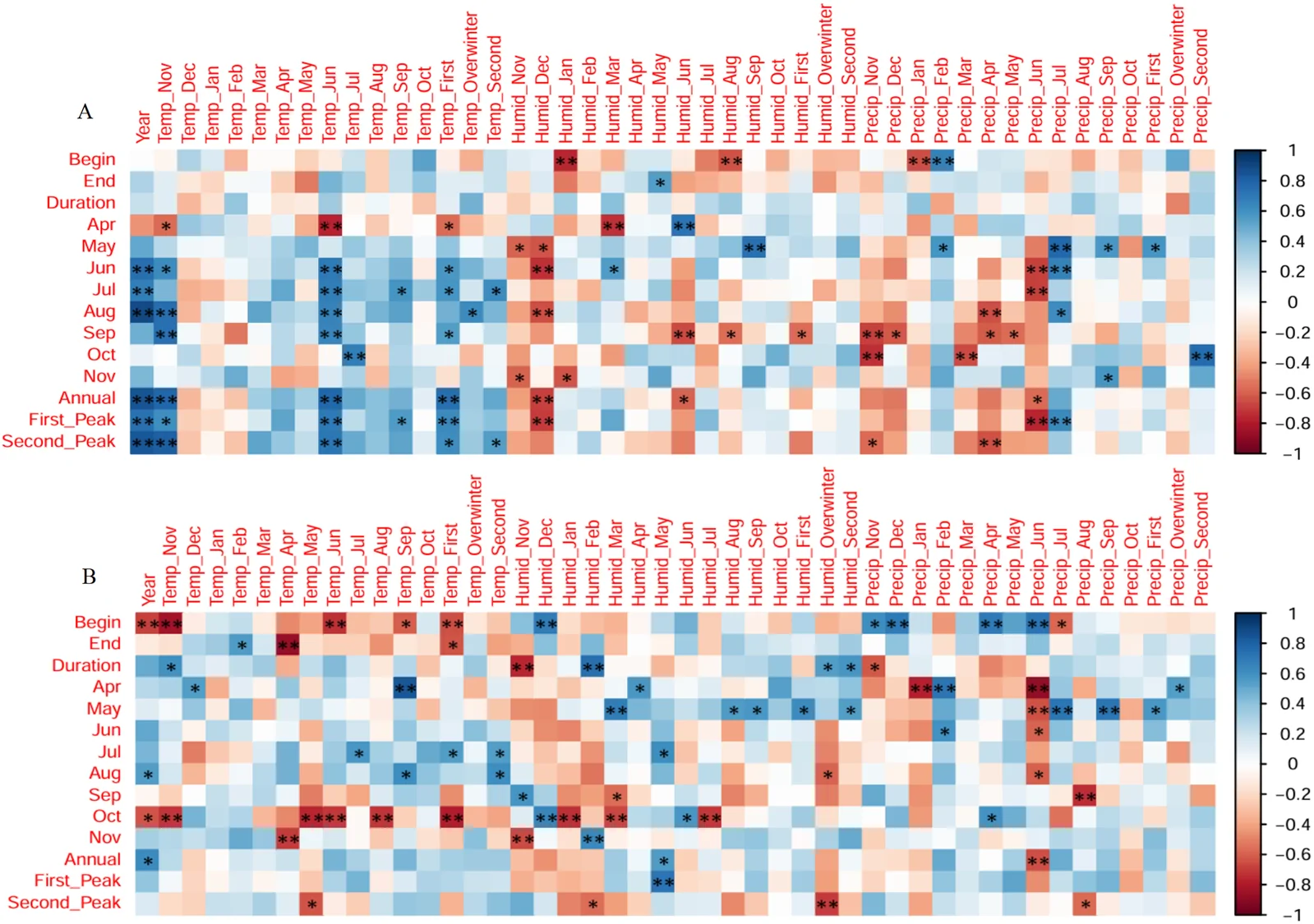
Spearman rank correlation matrices between lady beetle metrics and meteorological variables for *Harmonia axyridis* (A) and *Propylea japonica* (B). Rows are catch and phenological metrics (see axis labels) and columns are meteorological variables, expressed as monthly values (temperature and humidity as means, precipitation as totals) and as seasonal aggregates with suffixes Overwinter (previous-November–March), First (April–July) and Second (August–October). Cell colour encodes Spearman’s r_s_ (blue = positive, red = negative; scale −1 to +1); * *P* < 0.1, ** *P* < 0.05.

#### Stepwise multiple regression

To identify the most parsimonious set of climatic predictors, stepwise multiple linear regression was applied to z-standardised annual catches (*n* = 10 per species). For *H. axyridis*, the final model retained four predictors: June mean temperature, May cumulative precipitation, previous-December mean relative humidity, and April precipitation. June temperature and May precipitation showed significant positive associations with catch, whereas previous-December humidity and April precipitation had negative coefficients that did not reach significance (Fig. 6A; predictor coefficients in the figure legend). The model accounted for a large proportion of in-sample interannual variance (adjusted *R*^2^ = 0.90; in-sample RMSE = 130.3 individuals).

**Figure 6:**
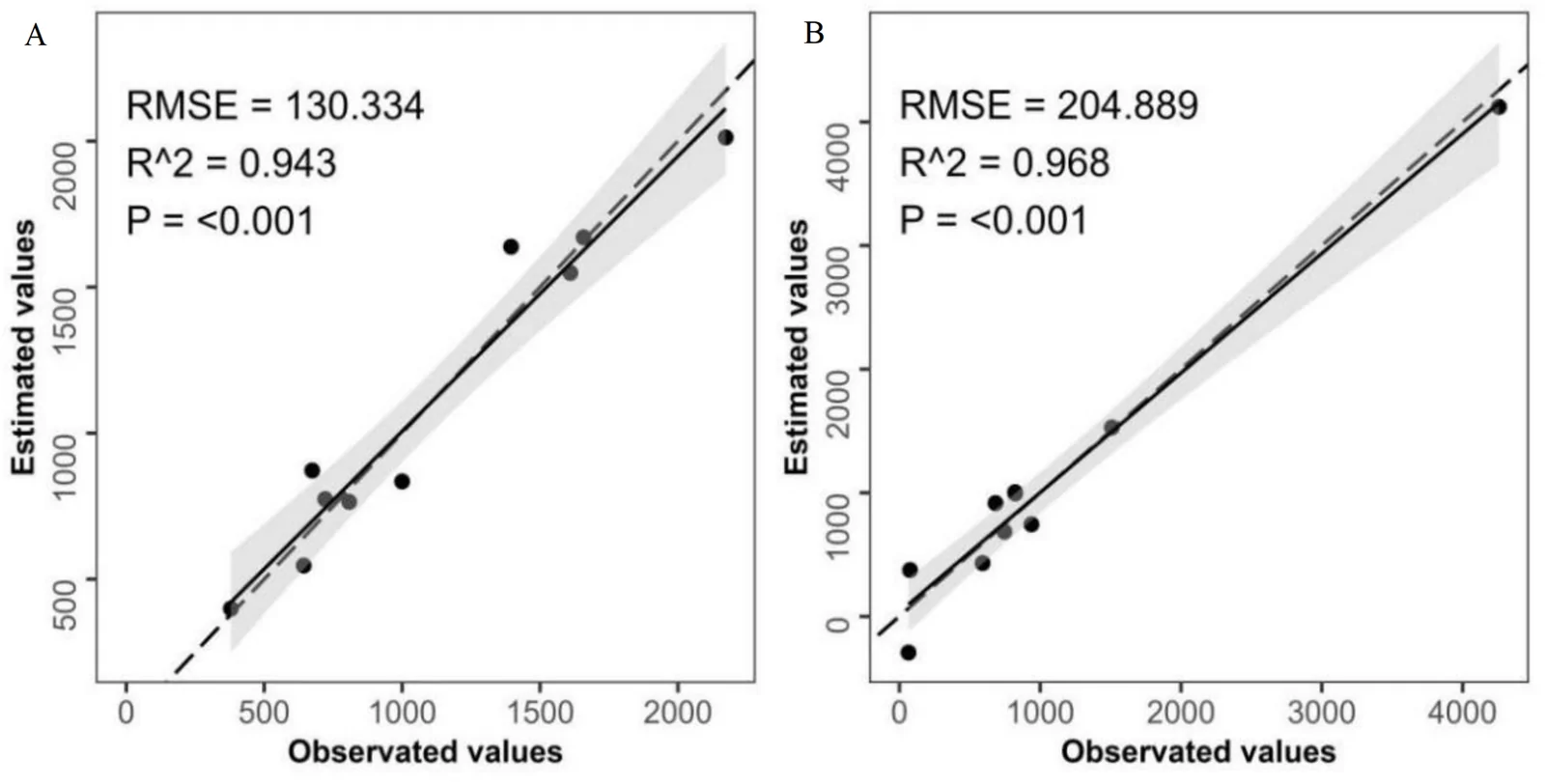
Observed *vs*. in-sample fitted annual light-trap catches from the final stepwise multiple linear regression models for *Harmonia axyridis* (A) and *Propylea japonica* (B). Solid line, regression fit with 95% confidence band; dashed line, 1:1. *R*^2^ and RMSE are in-sample metrics. (A) *H. axyridis*: June temperature (β = 0.90 ± 0.15, *P* = 0.002), May precipitation (β = 0.74 ± 0.18, *P* = 0.010), April precipitation (β = −0.42 ± 0.17, *P* = 0.058), previous-December humidity (β = −0.27 ± 0.13, *P* = 0.093). (B) *P. japonica*: May precipitation (β = 1.72 ± 0.17, *P* < 0.001), previous-November temperature (β = 0.92 ± 0.13, *P* < 0.001), September humidity (β = −0.49 ± 0.12, *P* = 0.011), April precipitation (β = −0.30 ± 0.13, *P* = 0.064).

For *P. japonica*, the final model likewise retained four predictors: May cumulative precipitation, previous-November mean temperature, September mean relative humidity, and April precipitation. May precipitation and previous-November temperature showed significant positive associations with catch, whereas September humidity had a significant negative association and April precipitation a negative coefficient that did not reach significance (Fig. 6B; predictor coefficients in the figure legend). This model yielded a higher in-sample fit (adjusted *R*^2^ = 0.94; in-sample RMSE = 204.9 individuals).

Notably, while May precipitation was the only shared predictor with a consistent positive effect, April precipitation was also retained in both models but exhibited a negative association.

### Predator–prey dynamics and phenological asynchrony

The 10-year mean dekadal profiles revealed a marked seasonal offset between predator and prey (Fig. 7). Aphid populations exhibited a bimodal pattern with a dominant spring peak (mid-April to late May) and a smaller autumn peak in September. In contrast, lady beetle activity peaked significantly later. For both species, the main occurrence period was concentrated from late May to mid-August and again from late September to early October.

**Figure 7:**
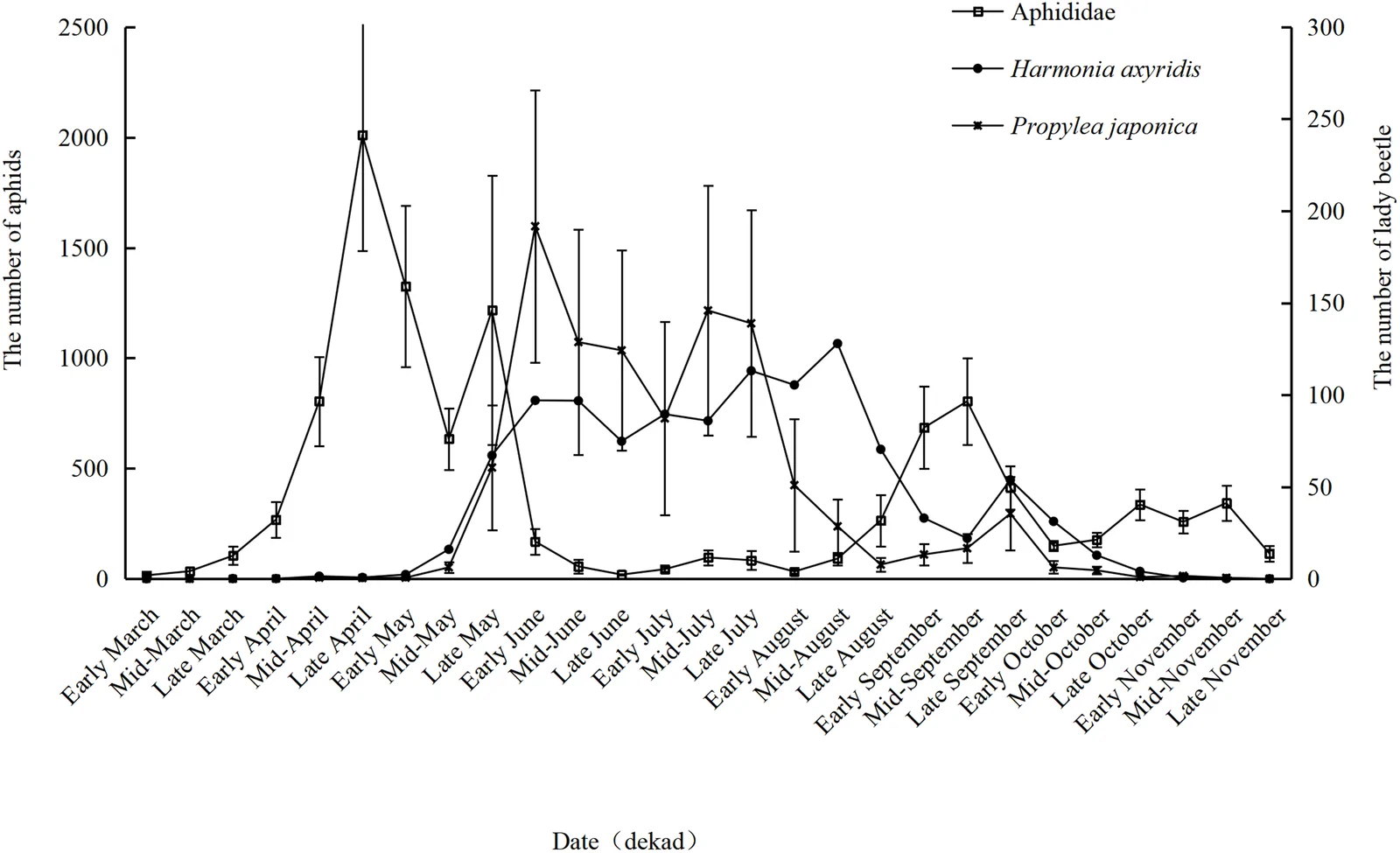
Ten-year mean dekadal (10-day) seasonal profiles of aphid and lady beetle catches. Aphid catches are plotted on the left axis and lady beetle catches (*H. axyridis*, *P. japonica*) on the right; symbols = 10-year means, error bars = ± SE (*n* = 10 yr).

Spring lags significantly exceeded autumn lags for both species. For *H. axyridis* (*n* = 10), the spring lag was 49.0 ± 9.2 d *vs*. 13.0 ± 15.7 d in autumn (paired t-test: t_9_ = 5.81, *P* < 0.001). For *P. japonica* (spring *n* = 8, autumn *n* = 5), the spring lag was 47.3 ± 10.6 d *vs*. 12.0 ± 8.4 d in autumn (Welch’s t-test: t_10_ = 6.65, *P* < 0.001). Across the 21 dekad positions of the 10-year mean seasonal profiles (Early April through Late October), both species were negatively correlated with aphid catches (Spearman; *H. axyridis*: r = −0.74, *P* = 0.0001; *P. japonica*: r = −0.63, *P* = 0.0023; Fig. 7): the mid-summer lady beetle peak coincided with the seasonal aphid trough, whereas the spring and autumn aphid peaks occurred when lady beetle catches were low. Collectively, these patterns—phenological lags, summer predator peaks under low prey density, and the absence of a positive interannual response—indicate a decoupling of predator activity from prey availability.

## Discussion

This decadal-scale study provides one of the first integrative assessments of the population dynamics, phenology, climatic correlates, and predator–prey relationships of the two dominant lady beetle species in the monsoon-influenced farmlands of central China. Three principal findings emerge, each corresponding to one of our *a priori* hypotheses. First, climatic factors—particularly May precipitation, June temperature, and previous-November temperature—were the dominant correlates of interannual population fluctuations for both species, while prey availability played a negligible role, supporting Hypothesis 1. Second, *P. japonica* advanced its first capture date by approximately 3.5 days per year while *H. axyridis* phenology remained stable, and the two species partitioned the growing season into complementary dominance windows, confirming Hypothesis 2. Third, predator peaks lagged behind aphid peaks by ~48 days in spring, summer predator maxima coincided with the seasonal aphid trough, and no positive interannual predator–prey association was detected, consistent with Hypothesis 3 that the numerical response is limited and abiotic forcing overrides trophic interactions. Below, we interpret these findings in the context of existing literature and discuss their implications for conservation biological control.

### Climate-driven population dynamics: asymmetric responses to shared climatic correlates

Both species responded to climatic variables, but through apparently different seasonal windows, reflecting divergent life-history strategies. The stepwise regression identified May precipitation as the only shared positive predictor (β = 0.74 for *H. axyridis*; β = 1.72 for *P. japonica*), while temperature predictors diverged: June temperature for *H. axyridis* and previous-November temperature for *P. japonica*. This asymmetry is not merely quantitative but qualitative—the two species are primarily associated with climatic signals operating in distinct seasonal windows.

The positive effect of May precipitation likely operates through habitat-mediated, rather than prey-mediated, pathways. In the wheat–maize rotation landscape of the Huang-Huai-Hai Plain, May corresponds to wheat grain-filling and harvest, marking the transition from dry spring to monsoon onset. Moderate May rainfall enhances soil moisture and field-margin vegetation, improving microhabitat conditions for overwintered adults emerging from diapause and for early-season reproduction (Bale et al., 2002; Nedvěd & Honěk, 2012). Crucially, our data decoupled this effect from aphid abundance—annual aphid catches showed no positive relationship with May rainfall. Conversely, the negative Spearman associations with June precipitation (both species ρ ≈ −0.6) reflect the destructive physical impact of heavy monsoon rains: intense rainfall causes direct mortality, suppresses flight activity, and reduces foraging efficiency (Hance et al., 2007; Thomson, Macfadyen & Hoffmann, 2010). The negative associations with April precipitation (both ρ ≈ −0.5) likely reflect a distinct mechanism: early-season storms physically dislodge small, freshly emerged adults from vegetation before they have accumulated sufficient lipid reserves, whereas June rains primarily suppress flight and foraging during the main reproductive period. The opposing signs of May (positive) *vs*. April and June (negative) precipitation effects underscore the non-linear, timing-dependent nature of rainfall impacts—a pattern well documented in tropical and subtropical systems (Wolda, 1988) but rarely quantified for temperate predators.

The species-specific temperature responses align with differences in overwintering ecology. *H. axyridis*, a highly dispersive polyphagous generalist with broad thermal tolerance, exploits diverse resources throughout the growing season (Koch, 2003; Roy et al., 2016). Its population size is therefore determined by conditions during the main reproductive period: warmer Junes accelerate development, increase voltinism, and extend the window for population growth (Osawa, 2000; Hodek, van Emden & Honěk, 2012). In contrast, *P. japonica* is a smaller, early-season specialist whose population trajectory is set before the growing season begins. The strong positive effect of previous-November temperature operates through overwintering survival: mild autumns reduce pre-diapause energy expenditure, allow longer foraging for lipid accumulation, and decrease cold-induced mortality during the early overwintering phase (Bale & Hayward, 2010; Sinclair et al., 2003). For *P. japonica*, which enters diapause earlier and in more exposed microhabitats than *H. axyridis* (Hodek, van Emden & Honěk, 2012), a warm November may substantially increase the proportion of adults surviving to reproduce the following spring. Such divergent climatic sensitivities have been documented in European coccinellid communities (Sloggett, 2021; Poutsma et al., 2008), and our results extend this pattern to the East Asian monsoon region, confirming the proposition raised in our Introduction that monsoon climate may impose stronger abiotic filtering than trophic interactions on predator population dynamics.

The high in-sample *R*^2^ values (0.90 and 0.94) should be interpreted cautiously given *n* = 10 and a 36-predictor candidate pool; they reflect descriptive fit rather than out-of-sample predictive power. Nevertheless, the consistency of May precipitation across both species (stepwise regression) confirms this variable captures a genuine climatic signal.

### Phenological advancement and temporal niche partitioning

The significant advancement of *P. japonica* first capture dates (−3.5 d yr^−1^), coupled with the absence of any directional trend in *H. axyridis* phenology, represents a differential phenological shift not previously documented for sympatric lady beetles in China. The magnitude (~35 days over 10 years) exceeds average rates for European insects (~2–5 d decade^−1^; Parmesan & Yohe, 2003) and is comparable to the most responsive taxa in meta-analyses (Thackeray et al., 2016). The mechanism likely involves the positive effect of previous-November temperature: as autumn warming progresses, overwintering adults accumulate thermal units earlier in spring, triggering earlier diapause termination and first flight (Bale et al., 2002). This is corroborated by the Spearman correlation showing previous-November temperature was negatively correlated with *P. japonica* first capture date.

The stability of *H. axyridis* first capture dates suggests this generalist may already emerge near the earliest phenologically possible date constrained by photoperiod or minimum thermal thresholds (Koch, 2003), or its spring phenology may be governed by migratory influx from southern source populations rather than local temperature (Osawa, 2000).

The temporal niche partitioning-*P. japonica* dominating June–July and *H. axyridis* August–October—establishes a seasonal succession that likely reduces interspecific competition and intraguild predation. Intraguild predation between these species has been demonstrated in laboratory studies, with *H. axyridis* larvae preying on *P. japonica* eggs and larvae (Yasuda et al., 2001). Temporal segregation may therefore represent an emergent coexistence mechanism (Snyder & Ives, 2001). The multi-peak phenology of *P. japonica* (early June, mid-July, late September), documented across a full decade, confirms and extends the shorter-term field observations documented in Chinese cotton and wheat systems (Ma & Liu, 1985; Gao, Li & Zhao, 2006; Ma et al., 2012). Strong interannual variability in peak number—from a single weak peak in 2019 to a sustained summer plateau in 2023—indicates this pattern is not a fixed programme but a plastic response to annual climatic conditions: adequate May rainfall supports the first peak through overwintered-generation reproduction, while subsequent peaks correspond to the first and second filial generations whose success depends on summer thermal conditions (Chen, Qin & Yun, 2009; Nedvěd & Honěk, 2012). Under continued warming, the progressive advancement of *P. japonica* phenology could increase temporal overlap with *H. axyridis*, potentially intensifying intraguild interactions (Roy et al., 2016).

### Predator–prey decoupling and limited numerical response

The persistent phenological decoupling between aphid and lady beetle peaks is a central ecological result. Spring aphid peaks were followed by predator peaks after approximately 48 days (both species; *P* < 0.001), while autumn lags were only ~12 days. Throughout midsummer, both lady beetles reached high activity levels precisely when aphid catches were at their seasonal minimum. The strong negative correlation between the 10-year mean seasonal profiles of aphids and lady beetles (*H. axyridis*: r = −0.74; *P. japonica*: r = −0.63) quantitatively confirms this asynchrony—a textbook signature of limited numerical response (Hemptinne, Dixon & Coffin, 1992; Dixon, 2000).

The ~48-day spring lag is mechanistically interpretable: the spring aphid peak (mid-April to late May) occurs during wheat heading–grain-filling, when aphid densities are high but transient. Coccinellid offspring from adults that aggregated on this pulse require 3–5 weeks to complete development (Kindlmann & Dixon, 1996); by the time they reach adulthood in late June–July, wheat has been harvested and the aphid resource has collapsed. The July–August predator peak thus reflects the predators’ own phenological programme, driven by temperature and photoperiod, rather than a reproductive response to the spring prey pulse (Hodek, van Emden & Honěk, 2012). The shorter autumn lag (~12 days) suggests tighter coupling, as autumn aphid populations on maize and soybean are more spatially stable and predator adults are already present in the landscape, requiring no generational turnover to respond (Winder et al., 2005). Nevertheless, even this shorter lag is insufficient to generate a positive numerical feedback at the population level.

The negative interannual correlations between predator and aphid catches (*H. axyridis*: ρ = −0.56; *P. japonica*: ρ = −0.26) admit several non-exclusive explanations. Years with high spring aphid abundance may coincide with climatic conditions (*e.g*., cool, dry springs) unfavourable for predator reproduction (Durant et al., 2007). Alternatively, high aphid densities may reflect low predator populations—a signature of predator limitation rather than prey limitation (Menge & Sutherland, 1987). Additionally, the suction-trap aphid index integrates all airborne aphid species at landscape scale, whereas light-trap lady beetle catches reflect local field-level activity; this scale mismatch may further attenuate any true positive predator–prey association (Harrington et al., 2007).

These findings align with the broader literature showing that abiotic forcing can override trophic interactions in systems subject to strong seasonal climatic forcing (Stenseth et al., 2002; Visser & Both, 2005; Both et al., 2009). The East Asian monsoon regime, with its pronounced wet–dry seasonality, may represent a particularly strong abiotic filter constraining predator populations independently of prey availability (Thomson, Macfadyen & Hoffmann, 2010), confirming our Hypothesis 3.

### Divergent population trajectories and community restructuring

The significant increasing trend in *H. axyridis* catches over the decade, contrasted with the non-directional fluctuations of *P. japonica*, suggests ongoing community restructuring in which the cosmopolitan generalist is gaining relative dominance. This pattern mirrors observations from Europe and North America, where *H. axyridis* has expanded dramatically (Brown et al., 2008; Roy et al., 2016). However, unlike the European context where *H. axyridis* is a recent invader, it has long been established in central China. The increasing trend likely reflects differential responses to regional warming rather than invasion dynamics: *H. axyridis* benefits directly from warmer growing-season temperatures (June temperature, April–July mean), whereas *P. japonica* depends on pre-winter conditions that have not shown the same directional trend—a direct consequence of the asymmetric temperature sensitivities identified above. The positive correlation between annual catches of the two species (r = 0.72) indicates that they still respond to shared drivers—most notably May precipitation—and the community is not yet undergoing competitive exclusion but rather a gradual shift in relative abundance driven by differential climate sensitivity (Sloggett, 2021).

### Implications for conservation biological control

Our findings carry a clear message: the efficacy of dominant natural enemies in monsoon-influenced agroecosystems may be more tightly coupled to climatic variability than to pest pressure. Conservation strategies focusing solely on habitat management to boost prey populations may prove insufficient if overarching climatic constraints are not considered (Landis, Wratten & Gurr, 2000; Gurr et al., 2017). The asymmetric sensitivity of the two predators implies that climate extremes may differentially decouple them, potentially eroding the temporal complementarity that underpins season-long pest suppression.

The temporal niche partitioning dictates that both species should be sustained: the early-season activity of *P. japonica* coincides with the aphid-susceptible period of maize and soybean, while the late-season dominance of *H. axyridis* provides sustained autumn pest suppression. Loss of either species would create a temporal gap in biological control coverage (Naranjo & Ellsworth, 2009). Management interventions should therefore target climatic buffering—for example, through overwintering refugia (hedgerows, beetle banks, leaf-litter habitats) that mitigate temperature extremes and reduce cold mortality for *P. japonica* (Nedvěd & Honěk, 2012). Operationally, degree-day models parameterised with previous-November and June temperature thresholds could provide predictive forecasts of *P. japonica* and *H. axyridis* emergence timing, respectively, enabling growers to time insecticide-avoidance windows to coincide with peak predator activity (Thomson, Macfadyen & Hoffmann, 2010; Hance et al., 2007). The positive interannual correlation between the two species (r = 0.72) further implies that May-precipitation-rich years simultaneously boost both predators, enhancing biocontrol services, whereas drought years suppress both—providing a basis for early-warning forecasts that allow pest management actions to be adjusted proactively.

### Limitations and future directions

Several caveats must be acknowledged. First, the single-site design limits spatial generality; multi-site monitoring across the Huang-Huai-Hai Plain is needed. Second, the 10-year duration provides limited statistical power (*n* = 10); the stepwise models should be regarded as exploratory identifiers of candidate climatic windows rather than predictive models. Third, light-trap catches index adult flight activity influenced by nightly weather conditions, rather than absolute field population density (Southwood & Henderson, 2000). Fourth, the suction-trap aphid index integrates all airborne aphid species at landscape scale, whereas lady beetle catches reflect local field-level activity; this scale mismatch may attenuate true predator–prey associations. Fifth, we did not account for potential confounding changes in pesticide use (particularly neonicotinoids) or landscape composition over the study period, both of which shifted in the Huang-Huai-Hai Plain between 2014 and 2023 and could independently affect predator and prey populations. Sixth, we did not directly measure alternative prey, floral resources, or interspecific interactions; these biotic factors may contribute to observed patterns but cannot be disentangled from climatic effects in this correlative framework.

Future research should prioritise: (i) multi-site monitoring networks across climatic gradients to test the spatial robustness of the phenological asymmetry documented here; (ii) experimental manipulation of overwintering conditions (*e.g*., warming enclosures) to test the causal link between November temperature and *P. japonica* survival and spring emergence; (iii) stable isotope or mark–recapture studies to determine whether early-season *H. axyridis* adults originate from local overwintering populations or long-distance immigration, thereby testing the migration hypothesis proposed for its phenological stability; and (iv) mechanistic population models integrating temperature-dependent development, precipitation-driven mortality, and prey dynamics to move from correlative associations toward predictive understanding (Hance et al., 2007; Thomson, Macfadyen & Hoffmann, 2010).

## Conclusions

This decade-long study demonstrates that climatic factors—particularly May precipitation, June temperature, and previous-November temperature—are the dominant correlates of interannual population dynamics for the two most abundant lady beetle species in central Chinese farmlands, and that these species respond asymmetrically through apparently different seasonal windows. The significant phenological advancement of *P. japonica* and the stable phenology of *H. axyridis* are reshaping temporal niche partitioning, with potential consequences for interspecific interactions under continued warming. Most critically, the comprehensive decoupling of predator activity from aphid availability—at both interannual and seasonal scales—indicates a limited numerical response. This finding strongly suggests that abiotic forcing overrides trophic interactions in this monsoon-governed system. These findings challenge prey-centric paradigms of conservation biological control and argue for a paradigm shift: sustaining natural enemy efficacy under climate change will require integrating long-term climatic monitoring, overwintering habitat management, and predictive phenology models into pest management frameworks, rather than relying solely on prey-driven habitat enhancement.

## Supplemental Information

10.7717/peerj.21748/supp-1 Supplemental Information 1 Data on lady beetles captured under black-light traps from 2015 to 2024. Daily and annual catch of two ladybugs, annual and dekad catch of aphids, and daily meteorological data of the local area from 2015 to 2024.

## Additional Information and Declarations

### Data Availability

The following information was supplied regarding data availability:The data is available in the Supplemental File.

## References

[ref-18] Atlihan R, Chi H (2008). Temperature-dependent development and demography of scymnus subvillosus (Coleoptera: Coccinellidae) reared on hyalopterus pruni (Homoptera: Aphididae). Journal of Economic Entomology.

[ref-1] Bale JS, Hayward SAL (2010). Insect overwintering in a changing climate. Journal of Experimental Biology.

[ref-2] Bale JS, Masters GJ, Hodkinson ID, Awmack C, Bezemer TM, Brown VK, Butterfield J, Buse A, Coulson JC, Farrar J, Good JEG, Harrington R, Hartley S, Jones TH, Lindroth RL, Press MC, Symrnioudis I, Watt AD, Whittaker JB (2002). Herbivory in global climate change research: direct effects of rising temperature on insect herbivores. Global Change Biology.

[ref-3] Both C, Bouwhuis S, Lessells C, Visser M (2006). Climate change and population declines in a long-distance migratory bird. Nature.

[ref-4] Both C, van Asch M, Bijlsma RG, van den Burg AB, Visser ME (2009). Climate change and unequal phenological changes across four trophic levels: constraints or adaptations?. Journal of Animal Ecology.

[ref-5] Boukal DS, Bideault A, Carreira BM, Sentis A (2019). Species interactions under climate change: connecting kinetic effects of temperature on individuals to community dynamics. Current Opinion in Insect Science.

[ref-6] Brown PMJ, Adriaens T, Bathon H, Cuppen J, Goldarazena A, Hagg T, Kenis M, Klausnitzer BEM, Kovar I, Loomans AJM, Majerus MEN, Nedved O, Pedersen J, Rabitsch W, Roy HE, Ternois V, Zakharov IA, Roy DB (2008). *Harmonia axyridis* in Europe: spread and distribution of a non-native coccinellid. BioControl.

[ref-7] Chen J, Qin QJ, Yun ZH (2009). Effects of temperature on experimental population growth and development of *Propylea japonica*. Journal of Agricultural University of Hebei.

[ref-33] Comont RF, Roy HE, Harrington R, Shortall CR, Purse BV (2014). Ecological correlates of local extinction and colonisation in the British ladybird beetles (Coleoptera: Coccinellidae). Biological Invasions.

[ref-8] Dixon AFG (2000). Insect predator-prey dynamics: ladybird beetles and biological control.

[ref-9] Durant JM, Hjermann DØ, Ottersen G, Stenseth NC (2007). Climate and the match or mismatch between predator requirements and resource availability. Climate Research.

[ref-10] Gao XH, Li FY, Zhao Q (2006). Population dynamics and spatial distribution pattern of *Propylea japonica* in cotton fields. China Plant Protection.

[ref-11] Gurr GM, Wratten SD, Landis DA, You M (2017). Habitat management to suppress pest populations: progress and prospects. Annual Review of Entomology.

[ref-12] Hance T, van Baaren J, Vernon P, Boivin G (2007). Impact of extreme temperatures on parasitoids in a climate change perspective. Annual Review of Entomology.

[ref-13] Harrington R, Clark SJ, Welham SJ, Verrier PJ, Denholm CH, Hulle M, Maurice D, Rounsevell MD, Cocu N (2007). Environmental change and the phenology of European aphids. Global Change Biology.

[ref-14] Hemptinne J-L, Dixon AFG, Coffin J (1992). Attack strategy of ladybird beetles (Coccinellidae): factors shaping their numerical response. Oecologia.

[ref-16] Hodek I, van Emden HF, Honěk A (2012). Ecology and behaviour of the ladybird beetles (Coccinellidae).

[ref-17] Honěk A, Dixon AFG, Soares AO, Skuhrovec J, Martinkova Z (2017). Spatial and temporal changes in the abundance and compostion of ladybird (Coleoptera: Coccinellidae) communities. Current Opinion in Insect Science.

[ref-19] Kindlmann P, Dixon AFG (1996). Population dynamics of a tree-dwelling aphid: individuals to populations. Ecological Modelling.

[ref-20] Koch RL (2003). The multicolored Asian lady beetle, *Harmonia axyridis*: a review of its biology, uses in biological control, and non-target impacts. Journal of Insect Science.

[ref-21] Landis DA, Wratten SD, Gurr GM (2000). Habitat management to conserve natural enemies of arthropod pests in agriculture. Annual Review of Entomology.

[ref-22] Ma XY, Liu Z (1985). Population dynamics of *Propylea japonica* in cotton fields. Plant Protection.

[ref-23] Ma G, Men X, Du X, Dai M (2012). Species composition and dynamics of predatory arthropods in wheat-cotton intercropping fields in western Shandong. Plant Protection.

[ref-24] Macaulay EDM, Tatchell GM, Taylor LR (1988). The Rothamsted Insect Survey ‘12-metre’ suction trap. Bulletin of Entomological Research.

[ref-25] Menge BA, Sutherland JP (1987). Community regulation: variation in disturbance, competition, and predation in relation to environmental stress and recruitment. The American Naturalist.

[ref-26] Naranjo SE, Ellsworth PC (2009). Fifty years of the integrated control concept: moving the model and implementation forward in Arizona. Pest Management Science.

[ref-27] Nedvěd O, Honěk A, Hodek I, van Emden HF, Honěk A (2012). Life histories and phenology. Ecology and Behaviour of the Ladybird Beetles (Coccinellidae).

[ref-28] Osawa N (2000). Population field studies of the aphidophagous ladybird beetle *Harmonia axyridis* (Coleoptera: Coccinellidae): resource tracking and population characteristics. Population Ecology.

[ref-29] Parmesan C, Yohe G (2003). A globally coherent fingerprint of climate change impacts across natural systems. Nature.

[ref-30] Poutsma J, Loomans AJM, Aukema B, Heijerman T (2008). Predicting the potential distribution of the harlequin ladybird *Harmonia axyridis* in Europe using the CLIMEX model. BioControl.

[ref-31] Ren SX, Wang XM, Pang H, Peng ZQ, Zeng T (2009). Colored pictorial handbook of ladybird beetles.

[ref-51] R Core Team (2023). R: a language and environment for statistical computing.

[ref-32] Roy HE, Brown PMJ, Adriaens T, Berkvens N, Borges I, Clusella-Trullas S, Comont RF, De Clercq P, Eschen R, Estoup A, Evans EW, Facon B, Gardiner MM, Gil A, Grez AA, Guillemaud T, Haelewaters D, Herz A, Honek A, Howe AG, Hui C, Hutchison WD, Kenis M, Koch RL, Kulfan J, Lawson Handley L, Lombaert E, Loomans A, Losey J, Lukashuk AO, Maes D, Magro A, Murray KM, San Martin G, Martinkova Z, Minnaar IA, Nedved O, Orlova-Bienkowskaja MJ, Osawa N, Rabitsch W, Ravn HP, Rondoni G, Rorke SL, Ryndevich SK, Saethre MG, Sloggett JJ, Soares AO, Stals R, Tinsley MC, Vandereycken A, van Wielink P, Viglášová S, Zach P, Zakharov IA, Zaviezo T, Zhao Z (2016). The harlequin ladybird, *Harmonia axyridis*: global perspectives on invasion ecology. Biological Invasions.

[ref-35] Sinclair BJ, Vernon P, Klok CJ, Chown SL, Sinclair BJ, Vernon P (2003). Insects at low temperatures: an ecological perspective. Insect Survival in the Cold.

[ref-34] Sloggett JJ (2021). Aphidophagous ladybirds (Coleoptera: Coccinellidae) and climate change: a review. Insect Conservation and Diversity.

[ref-36] Snyder WE, Ives AR (2001). Generalist predators disrupt biological control by a specialist predator. Ecology.

[ref-37] Southwood TRE, Henderson PA (2000). Ecological methods.

[ref-38] Stenseth NC, Mysterud A, Ottersen G, Hurrell JW, Chan KS, Lima M (2002). Ecological effects of climate fluctuations. Science.

[ref-39] Thackeray SJ, Henrys PA, Hemming D, Bell JR, Botham MS, Burthe S, Helaouet P, Johns DG, Jones ID, Leech DI, Mackay EB, Massimino D, Atkinson S, Bacon PJ, Brereton TM, Carvalho L, Clutton-Brock TH, Duck C, Edwards M, Elliott JM, Hall SJG, Harrington R, Pearce-Higgins JW, Hoye TT, Kruuk LEB, Pemberton JM, Sparks TH, Thompson PM, White I, Winfield IJ, Wanless S (2016). Phenological sensitivity to climate across taxa. Nature.

[ref-40] Thomson LJ, Macfadyen S, Hoffmann AA (2010). Predicting the effects of climate change on natural enemies of agricultural pests. Biological Control.

[ref-41] Visser ME, Both C (2005). Shifts in phenology due to global climate change: the need for a yardstick. Proceedings of the Royal Society B: Biological Sciences.

[ref-42] Walther GR, Post E, Convey P, Menzel A, Parmesan C, Beebee TJC, Fromentin JM, Hoegh-Guldberg O, Bairlein F (2002). Ecological responses to recent climate change. Nature.

[ref-43] Winder L, Alexander CJ, Holland JM, Symondson WOC, Woolley C (2005). Predator activity and spatial pattern: the response of generalist carabids to their aphid prey. Journal of Animal Ecology.

[ref-44] Wolda H (1988). Insect seasonality: why?. Annual Review of Ecology and Systematics.

[ref-45] Yang L, Zeng Y, Xu L, Liu B, Zhang Q, Lu Y (2018). Change in ladybeetle abundance and biological control of wheat aphids over time in agricultural landscape. Agriculture, Ecosystems & Environment.

[ref-46] Yasuda H, Kikuchi T, Kindlmann P, Sato S (2001). Relationship between attack and escape rates, cannibalism and intraguild predation in larvae of two predatory ladybirds. Journal of Insect Behavior.

[ref-47] Yu GY (2010). The subfamily coccinellinae of China.

